# Converting Hybrid Potato Breeding Science into Practice

**DOI:** 10.3390/plants12020230

**Published:** 2023-01-04

**Authors:** Michiel E. de Vries, James R. Adams, Ernst-jan Eggers, Su Ying, Julia E. Stockem, Olivia C. Kacheyo, Luuk C. M. van Dijk, Pawan Khera, Christian W. Bachem, Pim Lindhout, Edwin A. G. van der Vossen

**Affiliations:** 1 Solynta, Wageningen 6703 HA, The Netherlands; 2 Institute of Biometris, Mathematical and Statistical Methods, Wageningen University and Research, 6700 HB Wageningen, The Netherlands; 3 Laboratory of Plant Breeding, Wageningen University & Research, Wageningen 6708 PB, The Netherlands; 4 Centre for Crop Systems Analysis, Wageningen University and Research, Wageningen 6700 AK, The Netherlands

**Keywords:** *Solanum tuberosum* L., potato breeding, F_1_ hybrid, plant breeding system, true potato seed, variety release

## Abstract

Research on diploid hybrid potato has made fast advances in recent years. In this review we give an overview of the most recent and relevant research outcomes. We define different components needed for a complete hybrid program: inbred line development, hybrid evaluation, cropping systems and variety registration. For each of these components the important research results are discussed and the outcomes and issues that merit further study are identified. We connect fundamental and applied research to application in a breeding program, based on the experiences at the breeding company Solynta. In the concluding remarks, we set hybrid breeding in a societal perspective, and we identify bottlenecks that need to be overcome to allow successful adoption of hybrid potato.

## 1. Introduction

Research on diploid potato is thriving. The challenge now is to translate the fruits of this research into practical breeding programs that result in varieties that farmers wish to use and are beneficial for end-users. Hybrid breeding is the technology of choice for plant improvement due to joint benefits to farmer and commercial stakeholders. Hybrid breeding provides farmers a uniform crop with superior performance across multiple traits while attracting commercial breeders with the incentive of intellectual property protection and a highly effective system for achieving long-term genetic gains [1]. Recently, Bradshaw described the theoretical background, with emphasis on quantitative genetics issues that drive decisions in a hybrid potato breeding program [2]. Here, we address the components of a hybrid breeding program based on the experiences of a commercial breeding company, Solynta. Hybrid breeding programs are typically partitioned into several smaller components with specific trait targets. This generally manifests in a bifurcation into (1) parent line development and (2) hybrid assessment programs. The former is mainly aimed at amassing alleles beneficial to complex traits, stacking of resistances through back-cross programs, and selection of highly heritable consumer/market traits, with the latter primarily focused on identifying the best parental combinations, along with evaluating yield stability and assessing specific regional adaptability. Thus, this dispersal of breeding targets over multiple stages and cycles increases ease of selection for quantitative trait improvement in exchange for the greater system complexity in hybrid breeding program design [3].

**Figure 1:**
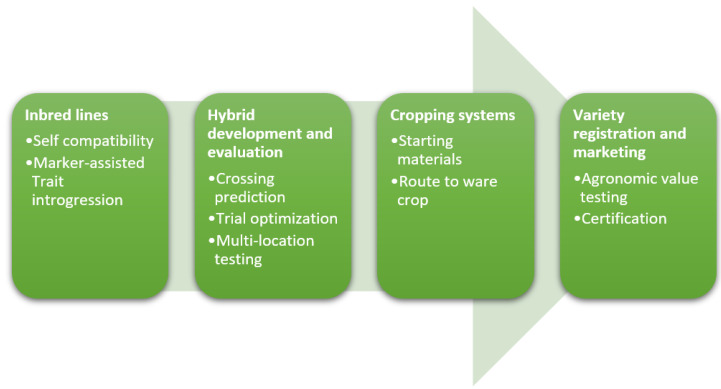
Main components of a hybrid breeding potato breeding program. From development of inbred lines to the release of a commercial product. In the first step inbred lines are developed, then hybrids are created, to arrive at a markable hybrid. These products are registered as varieties.

In this chapter we describe the different components that are needed for a successful commercial hybrid breeding program (Figure 1). They follow the trajectory from applied research to commercial product development. High quality inbred lines form the basis. These contain fixed traits of interest that can be combined to arrive at hybrids. Hybrid development and evaluation for different market segments is the next important phase. In case of Hybrid True Potato Seed (HTPS), the starting material is different from the traditional seed tubers, giving rise to changes in the cropping system. To be able to deliver products to the farmer, new varieties need to be registered and certified for marketing. Below we discuss the state-of-the-art and indicate where the gaps remain.

## 2. Inbred Line Development

A breeding program starts with a germplasm pool. From this pool diploid parent lines for hybrids are developed by an inbred line development process. The initial challenges for developing inbred lines center around developing self-compatible genotypes at the diploid level and overcoming inbreeding depression.

### 2.1. Germplasm

Breeding at the diploid level in potato starts with crossable genetic resources with ample genetic variation. From this gene-pool, inbred lines are developed. Variation can be sourced from diploid wild and cultivated sources [4], or from (commercial) tetraploids, from which di-haploids can be extracted [5]. For successful hybrid breeding, fertile, vigorous and homozygous inbred lines are needed. Both male and female sterility occur commonly in diploid potato germplasm [6]. In Lindhout et al., an overview of the initial genetic resources with which Solynta’s breeding program started, is described [7]. While the number of ‘founders’ used was limited, the observed genetic diversity was large. This diversity has since been illustrated by comparing genome sequences of heterozygous diploids and tetraploids [8,9,10]. Genetic variation that is not available in the founders can readily be incorporated into the diploid breeding program and therefore the founder materials need not contain all required traits. Changing market requirements and growing conditions are likely to require continuous revisions of breeding goals far into the future.

### 2.2. Self-Compatibility in Diploid Potato

For the development of diploid potato inbred lines, efficient self-fertilization is an essential trait. Many diploid wild potato relatives and landraces are self-incompatible (SI) [11], although, notably, within SI species, rare examples of self-compatible (SC) individuals occur [12,13]. This SI in tuber bearing Solanum is caused by an active gametophytic self-incompatibility (GSI) system that inhibits self-fertilization by arresting self-pollen tube growth in stylar tissues. The F-box proteins involved can recognize a wide variety of S-RNases except the one native to that allele, resulting in a collaborative non-self recognition system that allows cross-fertilization among a substantial portion of the germplasm [14]. In contrast, Hosaka & Hanneman found a self-compatible line in a *Solanum chacoense* accession [15]. They generated an F_1_ population segregating for self-compatibility, and mapped a gene that they named the S-Locus Inhibitor (*Sli*) to chromosome 12 [16]. More recently, Clot et al. used K-mer based bulked segregant analysis to map the *Sli* gene to more precise interval on chromosome 12 and showed that the *Sli* gene is present in a wide range of potato germplasm [17].

Although the *Sli* gene was successfully introduced in the breeding program, the causal gene model was not yet identified [18]. Thus, we set out to clone the *Sli* gene. Based on the collaborative non-self recognition model, we hypothesized that if the *Sli* gene is active in pollen, it would be gametophytically inherited since only pollen that contains the dominant allele would be able to self-fertilize. With this realization, we started surveying F_1_ populations for segregation of self-compatibility. F_1_ population 17SC11, derived from a cross between an SC mother and an SI father, showed clear segregation for self-compatibility as determined by fluorescence microscopy of pollinated styles and self-fruit and seed set. Genetic analysis in population 17SC11 revealed a highly significant QTL on chromosome 12, corresponding to the location identified earlier [16,19].

Next, we used a recombinant screening approach to narrow down the interval to only two genes and showed that one of these genes, encoding an F-box protein with a PP2-B10 lectine domain, is specifically expressed in pollen of SC lines. Sequence analysis of the SC haplotype of this F-box protein revealed a 533 bp MITE insertion in the promoter, which we hypothesize to lead to expression of the F-box protein in pollen. Finally, transgenic expression of the F-box gene with its native promoter including the 533 bp insertion converts SI lines to SC, whereas knock-out of this F-box protein in SC lines restores the self-incompatibility [20]. At the same time, Ma et al. identified the same gene in their diploid potato germplasm and showed that knock-out of this gene results in restoration of SI [21]. Furthermore, using yeast-two-hybrid and luciferase complementation assays they showed that the F-box protein can directly interact with 10 S-RNases out of a diverse set of 14, suggesting that the F-box protein encoded by the *Sli* gene acts as a wide spectrum S-RNase detoxifier [21].

Aside from the introgression of the *Sli* gene, multiple other routes to self-compatibility in diploid potato exist: self-compatible individuals of wild potato species may provide alternative self-compatibility genes whereas knock-down of the style expressed *HT-B* gene [22] or knock-out of the S-RNase [23,24] provide GM approaches towards self-compatibility.

### 2.3. Mapping Traits at the Diploid Level

When inbreeding populations have been developed, traits can be mapped and molecular markers can be developed. For a breeding program this is useful for marker-assisted selection. Ideally single gene traits can be followed with unique single nucleotide polymorphisms (SNPs), however, complex traits can be selected for using marker-based prediction models.

Many researchers have shown that traits that are selected for in tetraploid breeding programs, were also present in diploid potato germplasm [25,26,27]. Tuber shape was for instance mapped to the Ro locus with the round phenotype as the dominant effect [28], a finding confirmed by Meijer et al. [26] and Jansky and Endelman [25] in diploid inbred populations. Recently, the gene underlying the *Ro* locus was characterized and identified as *StOFP20* [29].

The current development of diploid hybrids involves recent and shallow pedigrees, with a limited number of founders. Within this context, alternative QTL detection methodologies should be considered to enable identification of relevant QTLs and characterize the founders of the pedigree [30]. Self-compatible germplasm allows to generate F_2_ and subsequent inbred generations, that facilitate mapping of recessive traits. For precise mapping of traits, high quality reference genomes are required. The release of our inbred line Solyntus provided an opportunity to develop such a reference genome [31]. More recently, an updated version of the DM genome was released [32], as well as two pan-genomes [9,10].

Although it goes too far to describe all possible trait mapping strategies here, from the above it becomes clear that all elements needed to conduct successful mapping studies routinely are present. With modern analysis tools, diploid self-compatible germplasm and well-assembled and annotated genomes, traits that can be phenotyped precisely, can be mapped.

### 2.4. Inbreeding Depression Hampers Line Development

Inbreeding depression in both plants and animal systems is thought to be primarily due to the exposure of recessive deleterious alleles as a result of becoming homozygous following self-pollination (dominance hypothesis) and to a lesser extent, by maintenance of advantageous heterozygous loci, where either allele as homozygotes, results in fitness penalties (overdominance hypothesis) [33,34]. Consecutive rounds of self-pollination increase homozygosity and allow selection of the fittest progeny that, presumably, will have an accumulation of advantageous alleles at all loci. While theoretically homozygosity is achieved following a simple mathematical rule, certain regions of the genome unpredictably appear to remain recalcitrant to further fixation [31,35]. This may be due to overdominance effects or regional repression of recombination. In order to achieve uniformity in hybrids, maximizing homozygosity of the elite lines is desirable. In addition, achieving a high level of diversity in different elite lines is also desirable to maximize the combinatorial heterosis effects in the hybrids. Recent work from several researchers does indicate that, while 100% homozygosity is difficult to achieve via self-pollination, it is possible to derive vigorous near-homozygous inbred lines that are highly suitable for hybrid breeding [36,37,38].

When a pool of self-compatible germplasm is developed, two complementary breeding strategies can be applied: (1) recurrent selection, aimed at maintaining beneficial alleles, and mainly focused on tuber yield, and (2) pedigree line development, focused on maintaining fertility. Alsahany et al. show the potential of recurrent selection in an early generation diploid pool [39]. However, in diploid potato, inbreeding depression is such a bottleneck [36], that pedigree-based selection for fertility and vigour is often a must before being able to select for other traits. Therefore, pedigree selection can be applied to reduce inbreeding depression to create a pool of fertile and vigorous lines. Then, recurrent selection can be applied to recombine positive alleles for complex traits.

In a hybrid breeding program, distinct pools are developed for a number of reasons. First, to exploit heterosis for complex traits (Section 3.1). Second, to be able to combine alleles in a hybrid that reside on the same locus, as is the case for some resistance genes in potato. Third, to manage traits for specific markets in an efficient way, for example processing and starch markets need high dry matter content germplasm, while table potato needs low. Chips potato need round potato, while the fries market needs long ones, therefore the material will need different alleles on the *Ro* locus. Although the extent of heterosis for complex traits has not yet been studied extensively, grouping and creating pools of inbred lines still makes sense for the other two reasons.

### 2.5. Trait Introgression

In any breeding program, the speed with which new traits are introduced is determining for its success. New traits can be introduced in a variety of ways, by phenotypic selection or by marker assisted selection. Another factor is when to select for which trait and what selection pressure is applied. Major determinants driving decisions in the breeding strategy are the genetic composition of a trait and whether the related phenotype expresses clearly under specific conditions. The genetic composition is one of the factors that determine the heritability, and traits with high narrow sense heritabilities (>0.7) can be selected for with higher accuracy. A high selection pressure can be applied with more than 96% of the individuals discarded. Application of SNP markers makes trait introgression and monitoring straight forward and cost effective. For potato, numerous molecular markers have been published, focusing mainly on pathogen resistances, e.g., Late Blight [40], Potato Cyst Nematode [41], and Wart disease [42]. However, due to potato’s large genetic variation it is not easy to identify SNPs that are generally applicable. There are different ways of application of marker assisted selection. One is a targeted approach to improve a limited number of valuable parent lines, or the aim can be to introduce the trait in the breeding pool. For the first case, in Section 2.6 an example is given. In the latter case, large scale screening at early generations is advised. Which strategy to choose when applying markers, differs per breeding program. There are a number of factors to weigh, most importantly is what is the value of bringing the trait to the market. When speed is most important, additional methods such as speed breeding, in which generation time is reduced and whole genome background screening, in which individual genotypes that already carry a large portion of the recurrent parents’ genome are selected, can be applied. On the other hand, when a specific resistance is required for all market segments, one may want to enrich the whole breeding pool for this gene. That can accomplished by using a resistant parent in breeding crosses, and then applying marker assisted selection at the seedling stage. This method is quite cost efficient, and can be applied to large numbers, but it takes more time before the trait reaches the market.

### 2.6. Marker Assisted Backcrossing, Example of Late Blight

**Figure 2:**
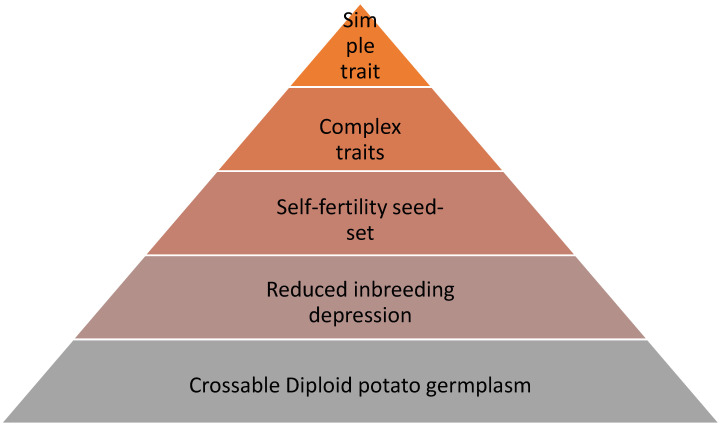
Schematic built-up of traits to be selected for subsequently in a diploid breeding program. The first pre-requisite is to have crossable diploid germplasm, then select for reduced inbreeding depression, then for seed-set to obtain large seed numbers, subsequently enrich the pool for positive alleles for complex traits, such as yield, and ultimately add simple traits through marker assisted selection.

After having obtained an inbred line, the quality of a line can be improved by adding market specific traits. Typically, this concerns resistance genes. Late blight has been identified as potato’s most important disease [43]. In potato and related germplasm, large numbers of resistance genes have been identified, and identification and cloning of new resistances has been accelerated by bioinformatic tools [44]. While at the same time it has become clear that for effective resistance in a crop, more than one resistance gene is needed [45]. This is due to the diversity of pathogen strains and adaptability of pathogens to resistances. To translate this knowledge and germplasm potential into value-adding varieties, marker assisted breeding is an efficient tool. Introgressing a single gene into an inbred line can be done using SNP makers. In Su et al., we demonstrated that when doing this in two of the parents simultaneously, a hybrid with two Late Blight resistances can be obtained in less than three years, using conventional back-crossing of the resistance donor, while using the parent line as recurrent parent [46]. However, the parent line in which the trait is introgressed should be valuable for other traits, preferably producing high-yielding hybrids (Figure 2).

### 2.7. Male Sterility

**Figure 3:**
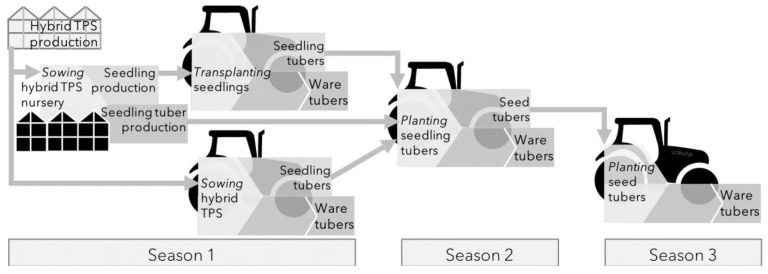
Routes to a ware crop, from van Dijk et al. 2021 [53]. In one, two or three seasons ware tubers can be produced. Transplanted seedlings were investigated by Van Dijk et al. [53,54,55]. Seedling tubers for ware production were investigated by Stockem et al., 2020 [56].

Male sterility is common in potato germplasm. In hybrid breeding, male sterility is used in many crops to facilitate open field seed production, resulting in lower production costs [1]. Depending on the exact route to a ware crop, discussed in Section 4 (Figure 3), true seed production cost may be an important factor. Therefore, it is important to note that the feasibility of male sterility for breeding has been investigated [47,48]. The most common type is cytoplastic male sterility (CMS), in potato there are six cytotypes described [49]. Discrimination between the different cytotypes is possible through DNA markers [50]. Recently a gene associated with tetrad sterility found in *S. stoloniferum* germplasm was identified [51]. To apply CMS efficiently in a breeding program, fertility restorer genes are important, to be able to use material freely as both male and female parent in crosses. Recently, Santaya et al., showed the presence of restorer genes in populations containing Late Blight resistance genes for different wild sources [52]. However, in the case of potato, where the tubers and not the seeds are the product, male sterile hybrids can be developed, which may have the additional advantage of leaving fewer seeds and reduce volunteers. The application of male sterility has large impact on the logistics of a breeding program, as male sterility needs to be maintained, which takes space and resources. One option could to swap cytoplasm of fertile tester lines, to generate a male sterile version through a backcross scheme.

From these studies, we can conclude that there is ample technical opportunity to apply CMS in hybrid potato breeding. The major driver for the decision whether to apply CMS is the balance between a more efficient seed production system and a more complex breeding system.

### 2.8. Inbred Line Development; Conclusions and Outlook

Recently, approaches to resolve inbreeding depression and genetic studies using self-compatible diploid materials have been published [26,36,37]. From these, we can conclude that the genetic prerequisites for developing diploid potato inbred lines have been met. The tools to develop inbred lines and map important traits are in place. It has been shown that enriching inbred lines with important single gene traits is possible. Clearly, all elements are available to develop inbred lines, as shown by example of the Solyntus line [31] and by Hosaka and Sanemoto [38].

Prioritizing among traits is important, therefore we have introduced in Figure 2 an overview of stacked properties that inbred lines in subsequent order should possess. At the bottom, it starts with crossable germplasm, then purging deleterious alleles against inbreeding depression. A reasonable self seed-set comes next, followed by complex trait with many minor effects, such as yield. Lastly, the mono-genic traits are considered, as they can be introduced by marker assistance.

New breeding techniques can add value to a hybrid breeding program by accelerated trait introgression. Witek et al. demonstrated that it is possible to introduce a Late Blight resistance gene into a diploid potato line by GMO-application [57]. Andersson et al. showed that CRISPR-based editing can be efficiently applied to knock-out genes in potato [58], while Perroud et al. confirmed it for the even more precise method of prime editing [59]. Currently, these technologies are stringently regulated in EU, but in the USA Richael showed that gene-edited potato varieties have a place in the market [60].

Now that pathways to resolve inbreeding depression and fertility as major bottlenecks have been demonstrated, the major remaining question will be, how to apply the presented selection tools at our disposal in the most efficient way.

## 3. Hybrid Development and Evaluation

It is one step to develop an inbred line and make hybrids for research purposes [37], it is another step to create a large-scale hybrid development program. Here, we discuss the most important elements concerning the hybrid creation and evaluation phase.

### 3.1. Crossing Strategies: Combining Abilities versus a General Breeding Value

The benefits of hybrid breeding do not come for free; along with the biological hurdles of establishing a new hybrid crop, there are other intricacies innate to hybrid breeding itself.

Hybrid breeding resolves this by dividing germplasm evaluation across multiple breeding moments, i.e., parental pool improvement, and hybrid test crossing, along with selection on multiple genetic targets (predominately, additive & dominant inheritance). This results in an increased efficiency for quantitative trait improvement, although the breeding program becomes more complex than a clonal or line based program.

The question of how a hybrid and its inbred parents are evaluated has resulted in the development of estimators like general and specific combining ability (GCA and SCA, respectively) [61]. Especially important is the ability to assess the performance of many parental lines (in the magnitude of hundreds or thousands), which is typical of a breeding program with more than one season in a year.

This problem of having to evaluate thousands of parental line combinations is dealt with in two ways: classification of inbred parents using a small subset or fixed set of crosses often called test-crosses. The aim is to assess the potential of new lines by crossing them with one or few known high-performing testers. Additionally, genetic models capable of predicting performance of hybrids based on pedigree, marker, or other relational data can be applied. Irrespective of the approach taken, a quantitative genetic description of your crop, traits of interest, and breeding strategy is bedrock in understanding a parental line’s value in a hybrid breeding program. Such an approach was undertaken in Adams et al. where we evaluated a sparse crossing set consisting of 800 hybrids in sandy and clay conditions soil and assessed for important quantitative traits behind economic yield of potato: yield, tuber number and size [62]. In this study we endeavored to estimate stable variances and covariances for GCA and SCA to assess the magnitude of additive and dominance gene action behind tuber phenotypes. We found that additive effects were the largest genetic effect behind all tuber phenotypes studied, but especially for tuber size. Dominance effects were found in all phenotypes but, at most, accounted for 14% of genetic variation. For all studied traits, we found encouraging heritabilities from 0.59 for yield up to 0.86 for tuber volume. The general combining abilities were on average twice as large as the specific combining abilities across traits. The importance of additive effects in this panel of hybrids shows that mid-parent value can be used as trusted indicator in hybrid evaluation. The genetical differences between the parents were large, and hence frequencies of dominant alleles for high yield were probably less than 0.5. Implications of this study not only suggest that GCA is a worthwhile target for selection in valuable tuber phenotypes, but more broadly, that evaluation of parents and their combinations, a core tenant of the hybrid schema, is a worthwhile effort in hybrid potato.

### 3.2. Hybrid Evaluation

When test crosses are made between inbred lines, it is important to evaluate the performance of the hybrids to determine the potential of the inbred lines. This evaluation is done in field trials, in which the test-hybrids are being benchmarked against existing commercial cultivars. These commercial cultivars typically are grown from seed tubers, while the test-hybrids are produced as true seeds. To avoid confusion over the terminology of various type of potato materials this paper refers to seedling tubers, as the product of a plant grown from TPS. In its consecutive season a planted seedling tuber could either bear seed or ware tubers. Seed tubers -used as starting material of a new potato crop- could, in this paper, originate from either seedlings, seedling tubers or a different type of tuber starting materials [63,64,65,66]. Ware potatoes, materials which are meant for consumption or any type of industrial use, could origin from any type of potato starting material. To make a good comparison, the starting material should be as similar as possible, therefore, we first produce seedling tubers in one field season. In the subsequent season, the test-hybrids are grown in replicated field trials together with commercial cultivars.

These trials are done in several locations, and in Stockem et al. we evaluated the performance and the environmental effects on yield and yield components between diploid hybrids and commercial cultivars [56]. Besides that, we looked at the contribution of different yield components to total yield, and how that differed between the hybrids and the commercial cultivars.

Yield was affected by environment and by genotype and there was a genotype × environment (G × E) interaction. On average, the diploid hybrids had a yield between 16 and 52 t/ha, for the commercial cultivars that was between 52 and 101 t/ha. Thus, the hybrids grown from seedling tubers showed a yield potential comparable with commercial cultivars with the highest yielding hybrids at the same level as the lower yielding commercial cultivars. Dry matter percentage (DM%) is an important trait for chips and frying cultivars. For the diploid hybrids DM% was between 16 and 21%, which is in range with commercial cultivars. Additionally, tuber shape is an important trait for fry and chips production. Since tubers need to be suitable for mechanized processing machines, the shape also needs to be as stable as possible. The same range in shape from round to long tubers that was present in commercial cultivars was found in the diploid hybrids. To compare the stability of tuber shape of individual tubers between cultivars, we calculated the coefficient of variation (CV). On average, hybrids had a CV of 20% and commercial cultivars 17%. The most stable hybrids showed overlap with the least stable commercial cultivars.

### 3.3. Field Trial Techniques

The accuracy of field trials is always affected by random variation at field and plot level. For example, nutrient distribution or soil particle size can vary in the field, leading to unexplained variation in the results. Furthermore, neighboring plots can affect each other by competing for resources. When we evaluate the performance of new hybrids in field trials, it is important that differences that are measured are the result of different genetics and not of field variation. A way to decrease random variation in field trials is to adjust plot dimensions, with larger plots generally resulting in lower variation. The plot shape that should be used depends on the field variation. In a homogeneous field square plots can be used, while in a heterogeneous field rectangular plots with most plants in the direction of the largest field variation are advantageous. In breeding trials such as described in Section 3.2, plot size is an important decision. Using large plots cost more resources that also could be used to evaluate more genotypes, and plots should be large enough to select superior genotypes. In Stockem et al. we analyzed the effect of plot dimensions on error variation for different traits, and we provide equations to estimate the expected variation for different plot dimensions [66].

Two field trials were performed (in 2017 and 2018) in which four diploid hybrids and one commercial cultivar (Hermes) were grown. We used plot sizes of 90 plants, divided over 6 ridges with 15 plants per ridge, and three replicates. Harvesting each plant individually allowed us to analyze the variation for all plot size up to 90 plants. We calculated the error variance as the least significant difference as a percentage of the trait mean (LSD%). This LSD% represents the minimal difference between two trait means to be significantly different from each other. Equations to estimate the LSD% were made for several important yield and quality traits: tuber weight, tuber count, tuber shape, tuber volume, and as for within-trait variation the standard deviation of tuber shape and volume. Increasing the plot size led to decrease in LSD% for all traits, while the LSD% value was different between traits.

When planning a field trial, breeders can use the equations from Stockem et al. to determine the plot size, which is needed to attain the desired level of precision for a certain trait [66]. For example, plots of 40 plants have an LSD of 15%.

### 3.4. Conclusion Hybrid Development and Evaluation

As potato has entered the hybrid breeding era, the challenges associated with hybrid breeding have become a priority. Choice of parents for a hybrid, creation of parent pools, and assessment of G × E of yield related traits are among the most important ones. Here, we show that we have developed a program that generates a large number of hybrids, which are evaluated for traits that can only be evaluated in the field.

One of the most important outstanding issues is prediction of field traits, which can be phenotyped with confidence from a crop grown from a seedling tuber. As it can take two years to generate phenotypic information after the hybrid has been created for traits such as yield, dry matter and tuber size distribution, there is a need for faster methods. These could be derived traits that already can be phenotyped at the seedling stage, or genomic prediction models.

## 4. Cropping Systems

The introduction of hybrid breeding and the consequent use of HTPS as starting materials provides novel opportunities for potato crop production. Multiple cultivation pathways are available for both seed potato and ware potato production (Figure 3), and the feasibility of these pathways has been assessed [53]. Various propagules are generated during the progress of the pathways from HTPS to successive generations of seedling or seedling tubers, thereby further creating multiple and contrasting cropping systems within the pathways. The differences between the starting materials generated cause significant differences in crop growth and development as well as differences in various aspects of crop management required throughout the crop’s lifetime.

### 4.1. Development Scale adjusted for HTPS

An objective development scale for plant growth is needed for registration field tests, such as the Distinctness Uniformity and Stability (DUS) test, but also for recommendation of agronomic practices, e.g., optimal application timing of fertilizer. The BBCH scale, from Biologische Bundesanstalt, Bundessortenamt und CHemische Industrie, describes phenological stages of a crop by a decimal code and is widely used for this purpose. Hack et al., described an extended BBCH scale for both TPS and tuber- grown plants, which, however, proved limiting, due to the standardization of descriptions in various stages [67]. Kacheyo et al., augmented the BBCH scale for potato to also include plant growth from true seeds [65]. This was done to mostly provide detailed descriptions to the standardized descriptions of both TPS- and tuber-grown plants as described in the original scale. Morphological differences have been observed between tuber-grown and HTPS-grown plants, such as differences in the plant form and structure including types of branches, number of stems and types of stems as well as in below ground growth and development. The differences contribute to differences in crop growth and development between the contrasting starting materials as well as the general requirements for crop management [63,65,68]. A detailed scale was proposed, which could be utilized for both breeding and research purposes. The proposed scale can now be applied without any limitations based on starting material or the stage of plant growth.

### 4.2. Transplants

In recent years, the use of field transplanted, nursery-raised, hybrid potato seedlings has been tested as a potential alternative system for seed and ware potato production [54]. Ultimately, to obtain a highly productive transplanted crop, aspects of seedling production, field establishment and transplant field crop management need be considered and successfully executed [55]. These aspects include, but are not limited to, nursery climatic conditions and nursery management factors, field conditions at transplanting (soil and climatic conditions) and crop management factors, all of which, when successfully carried out, contribute to high productivity in transplanted crops [53]. We have reported on various aspects of crop management as well as the first results of yield for field transplanted crops of experimental hybrid genotypes. Additionally, we have reported effects of additional hilling, where no hilling, compared to additional hilling, resulted in larger tubers [53]. Transplanting densities of 25 and 50 plants m^−2^ resulted in optimum tubers yields in contrasting planting systems. We observed that tuber-size distribution was affected having an increasing share of large-sized tubers when planting density decreases [54]. Timing of transplants in spring and stage of the transplant are important new agronomic decisions that need to be taken. We investigated timing, and early planting, in March should be avoided due to high chance of lethal frost damage. The longer duration of the growing season was one observation from the transplanting moment study that became apparent. The timing of harvest was of some maturity types more important than the timing of -or conditions during- transplanting [55]. With these studies, we have shown the feasibility of the field transplanted systems. On the other hand, the transplanting system faces various challenges, and more importantly, the longer growing period in the field which influences tuberisation and tuber bulking as well as the timing of the moment of harvest [54].

### 4.3. Seedling-Tubers

The first generation of tubers produced from a seedling crop, can be used to produce a ware crop (Figure 3). As discussed under 4.1, a transplanted seedling crop is technically more demanding, and has a longer cycle. Therefore, in many conditions, a ware crop grown from seedling tubers, is a profitable production route. It was shown that it is possible to perform yield trials based on seedling tubers [39,64,69].

Yield levels from a seedling tuber-based crop are higher than from transplanted crop, from the same genotype, due to slower canopy closure of transplants at the start of the season. This coincides, in temperate regions, with the period of highest irradiation level, which aggravates the impact of slower growth in the beginning of the season. Transplanted crops also have a longer cropping cycle [55], which increases costs of production, compared to a seedling grown crop. Lastly, farmers are currently used to producing their potato crop from seed-tubers. Convenience and ease of use are factors that are critical for successful adoption but are often overlooked. Due to these reasons, we foresee that the ware crop will be tuber-based for the first generations of hybrid potato.

However, for a breeding scheme it is vital to determine the highest yielding hybrid as rapidly as possible. If this can be done from a transplant, without having to clonally propagate, then it will save at least one year in the breeding cycle. Whether there is a fixed relation between yield from the two different starting materials, or whether it is genotype dependent, remains an open question.

### 4.4. Conclusion Cropping Systems

Developing a cropping system for a whole new crop is a major challenge. The change in starting material has many implications, many of which still need to be investigated. We have taken the first steps in exploring what is possible, and what the best routes from true seed to a ware potato are. These routes to a ware crop will be context dependent and probably will change over time. Therefore, breeding companies need to be able to facilitate the growers by supplying technical support for an array of different environments. A major challenge will be how to disseminate the right information to the growers.

## 5. Variety Registration and Marketing

### 5.1. Regulatory Hurdles for Variety Registration

Development of new potato varieties by means of hybrid seeds is a new development and results in a new type of plant propagating material for the EU: Hybrid True Potato Seed Varieties (HTPS-varieties). Two regulatory barriers need to be resolved in order to fully exploit the benefits of this new development: first, distinctiveness, uniformity and stability (DUS)-testing for variety registration and Plant Variety Rights applications and, second, marketing and certification. The next step is to realize this potential by releasing HTPS varieties to potato farmers. The development of HTPS varieties for commercialization is well underway. Several HTPS varieties have been submitted for variety registration and protection in The Netherlands since 2020. To allow EU farmers and consumers to benefit from improved varieties, policies regarding variety registration, Plant Variety Rights and certification and marketing require adaptation.

For variety registration as well as for Plant Variety Rights applications a DUS-test is used, to assess the Distinctness, Uniformity and Stability of a new variety. The current Technical Questionnaire (TQ) for DUS-testing is based on clonal tuber-propagated potato varieties. This protocol cannot be applied in all detail for HTPS varieties, so adaptations are needed to the uniformity requirements, propagating materials and field testing protocols. This concerns DUS-testing for National/EU variety listing, as well as DUS-testing for applications to obtain National or EU Plant Variety Rights.

### 5.2. Certification

Once a variety is registered, Certification and Marketing of true seeds, seedlings and potato tubers from HTPS-varieties need regulation. Council Directive 2002/56/EC defines the quality requirements for plant propagating material of potatoes in the European Union. These requirements are based on vegetatively propagated clonal potato varieties (tubers). In a scenario that true potato seeds, potato seedlings and first-generation tubers produced from HTPS-varieties are used for further propagation, the marketing is currently not regulated in the EU.

The following regulatory gaps have been identified:

- Seed-tubers derived from HTPS plants need to be classified according to the existing tuber class certification scheme.
- Minimum requirements for germination, purity and health status of the true seeds should be defined.
- Provision of quality requirements and inspection guidelines of seedlings of HTPS varieties intended to be transplanted for the production of potato tubers.

The EU Temporary Experiment, is intended to provide answers on how and on what conditions to allow certification and marketing of potato tubers from HTPS-varieties in the EU. The Temporary Experiment ends in 2023 and should result in an update of Council Directive 2002/56/EC in 2024.

### 5.3. Plant Material Exchange

Additionally, the present policy to ban all imports from HTPS from outside the EU, hampers seed production outside the EU, potentially increasing seed costs and complicating global logistics. For North America, at present it is possible to import HTPS material for research purposes, but not in commercial quantities. However, a process to increase speed and flexibility has been initiated by US authorities.

### 5.4. Conclusion Variety Registration and Marketing

In conclusion, to make a sustainable cultivation of potato possible, the governments should consider adapting regulations regarding DUS-testing for variety registration as well as Plant Variety Rights applications and certification and marketing to avoid delay in access to improved potato varieties for European farmers and consumers.

## 6. Implementation

### 6.1. Application and Execution

In the previous sections, an outline of all elements needed to build a hybrid breeding program for a new crop was sketched. In this section, we want to show what is needed to combine these elements. Insight in many of the scientific challenges was generated using a reductionist approach, which has led to addressing individual problems. Now when considering a breeding program as a whole, two important aspects stand out that are typically not treated in scientific studies: first operational excellence and second product management. However, both aspects are vital for a mature hybrid breeding program [70].

Operational excellence in a breeding program means to execute the experiments according to plan and delivering results in time according to a pre-defined protocol. Breeding is about making decisions efficiently, and these decisions are being made based on information generated by a team. The goal is to purge out inferior alleles as fast as possible. As there are many people that need to collaborate, delivering according to expectations on quality and timing is the norm, reducing the time needed to analyse and interpret results. To make explicit among colleagues, what is needed when, development of a set agreements, often in the form of protocols facilitates smooth collaboration between and among teams. One example where this typically applies is in SNP marker-assisted breeding. Breeders want to discard plants as early possible, however DNA sampling in potato is still being done from green leaf tissue. A large number of actions will be performed before the decision of discarding or keeping a plant can be made. Seeds need to be sown and plants maintained. The plants are leaf sampled, the tissue shipped to the genotyping lab, DNA extracted, SNP-assayed, SNP data interpretated and quality controlled, before linking results back to plants. For all these steps correct labelling and a database to ensure track-and-trace of each sample are prerequisites. All of these steps, which involve a large number of people, working in different teams at different locations, need to be executed in a matter of days. Automation and robotization of essential steps is needed to be able to process large volumes of samples in little time. Breeders need to be able to trust this process, as they base their decision on it. Therefore, internal transparency and explicit elaboration of protocols is helpful. In the collaboration between people, clear definition of task and responsibilities increases mutual trust and clarity. The quality of a breeding program depends on the speed and quality of the execution of such a process. Breeding programs can for such cases learn from management processes from other industries.

### 6.2. Information Sharing and Product Management

**Figure 4:**
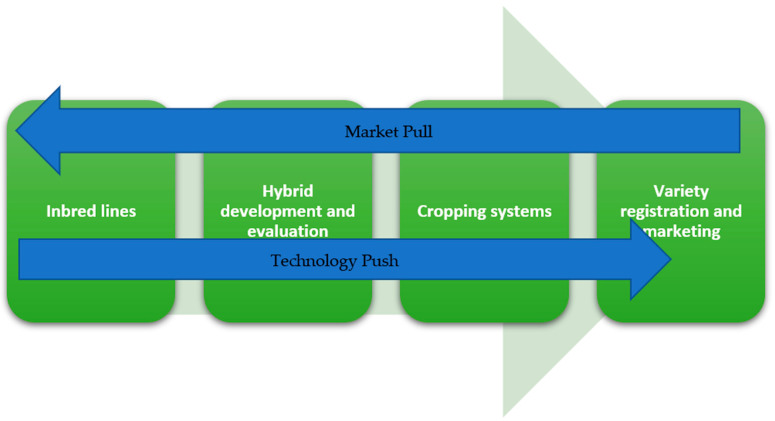
The two flows of information, Market pull and Technology push managed by product management. The product development pipeline in a breeding program runs from inbred lines to variety registration.

On top of excellence in execution of operations in a breeding program, there are two counteracting flows that needs to be managed in a breeding program. Evidently, when a large number of people collaborate to achieve the same goal, while working in different parts of the breeding program, information sharing and management of priorities becomes important. The two counteracting flows are market pull versus technology push (Figure 4). Market pull is based on external information on customer demands and preferences. Technology push is based on internal discoveries, not necessarily linked to current market demands. In the first stage of hybrid breeding, technology push was important. When developing a mature program, market pull becomes a more important driver for breeding strategies. Clearly, market demand needs to be made explicit, resulting in product profiles for specific markets. These can be overlayed with market size, to determine importance and resource allocation for a specific product profile. New discoveries need to be matched with existing product profiles, or development of new markets. For example, discovery of heat or cold tolerant genotypes can lead new potato growing environment, developing completely new markets. For HTPS, due to its logistic advantages, growing environments that are characterized by high transport costs and seed-tuber storage problems, are a new market opportunity, developed by technology push. On the other hand, the increase in demand by consumers and policy makers for potato varieties that need less crop protection agents is strong market pull to develop late blight resistant hybrids.

Setting the right strategy to allocate resources, based on information of both markets and discoveries, is vital for a breeding program. To efficiently and transparently collect and manage the information, decide and communicate the decisions, are the pivotal roles that product management plays in a breeding program.

## 7. Societal Context and Future Outlook

### 7.1. Hybrid Potato Breeding and Society

Innovations are developed by inventors, but the users ultimately determine their success. Successful uptake of an innovation is determined by whether it is accepted by society and whether it has added value for the user. The case of hybrid potato breeding has led to a number of studies [71,72] in which the question whether hybrid potato was acceptable for society and in what form was investigated. A number of potential scenarios were discussed by stakeholders from the potato value chain. These stakeholders were from private and public sector, representing farmers, consumers and civil society. The most important lessons to be drawn from these were for governments to provide answers how to facilitate an acceptable and equitable future for all stakeholders. Therefore, we reaffirm the advice of the Rathenau institute, that advises the Dutch government on new technologies and innovation. It states that development of a legislative framework, as discussed under 5.1, is important for adoption of HTPS [71].

Another aspect in which governments play an important role is making sure there is a diversity of genetic material available and accessible, and most importantly they can assure that a scientific basis for further development of the potato value chain is supported [73]. The role of government support is essential to further and deepen fundamental knowledge in the public space. Public funding from the USA [74], China [37] and The Netherlands (Holland Innovative Potato) have been of essential support to provide insight in fundamental scientific questions surrounding hybrid diploid potato breeding. The use and application of this knowledge can, as is the case with many crops, be left to a diverse set of private players. However, the investments in fundamental research are such that only very large private parties can afford it. If governments have a desire to support a diverse set of private players, then public funding of fundamental science is the best assurance. Although there has been support from public funding, a number of challenges that are outstanding are amongst the most difficult ones: yield stability under abiotic stress as a prominent example. Due to a climate that becomes more erratic, a society that demands a sustainable agriculture with minimal environmental impact, and farming incomes that are under pressure, stable yield is the holy grail.

The present commitment of two world leading potato processors, Simplot and AVEBE, to invest in hybrid breeding is encouraging. It reflects the realization that in the longer term this technology is needed to address the multi-faceted demands from food products.

### 7.2. Challenges Still to Tackle

In this review, we have listed the factors that are needed to allow a hybrid breeding program to function. We can conclude that our major challenges have shifted from purely scientific with a focus on genetics, now to registration and marketing, and to developing sturdy agronomic protocols for growing potatoes from seedlings and ultimately from true seeds.

With this we can point out specific challenges at three levels:

Protection of varieties and exchange of germplasm. Accelerated adjustments of policies will enable breeders to exchange germplasm, allowing faster genetic gain, and increase access to the best varieties for farmers. Protection of varieties increases certainty for breeders to obtain a fair return on investment.

Research and development of agronomic practices needs to be expanded to potato growing areas outside of Europe. In Africa and Asia, HTPS has huge potential to increase yield and food security. To realize this potential, it is needed to design, develop and disseminate knowledge on crop management.

Development of resilient yielding hybrids. Potato is very vulnerable to abiotic stresses, heat and drought being the most prominent ones. Research into development of traits to mitigate effects of abiotic stress is needed. Fundamental science is still needed in unravelling the interactions between tuber dynamics and the environment.
